# Arthrocentesis of Temporomandibular Joints—A Clinical Comparative Study

**DOI:** 10.3390/life14121594

**Published:** 2024-12-03

**Authors:** Marta Siewert, Rafał Pokrowiecki, Paweł J. Zawadzki, Zygmunt Stopa

**Affiliations:** 1 Department of Cranio-Maxillofacial Surgery, Oral Surgery and Implantology, Medical University of Warsaw, 02-005 Warsaw, Poland; martasiew@gmail.com (M.S.); kcst@wum.edu.pl (P.J.Z.); z.stopa@wp.pl (Z.S.); 2 Private Practice, 02-640 Warsaw, Poland

**Keywords:** temporomandibular, arthrocentesis, maxillofacial, TMJ, pain, hyaluronic acid

## Abstract

The objective of this study was to compare single-needle arthrocentesis with the conventional two-needle arthrocentesis, as well as the additional intracapsular injection of hyaluronic acid or platelet-rich fibrin. A total of 96 patients with established osteoarthritis (OA) (*n* = 48) or with internal de-arrangement (DD) (*n* = 48) were assigned single-needle arthrocentesis with distension of the joint or conventional two-needle arthrocentesis with or without intracapsular injection of the medication (hyaluronic acid (HA) or platelet-rich plasma (PRP)) performed every month over a period of 6 months. The maximum mouth opening and pain, as measured by the visual analog scale (VAS), were compared. Each group exhibited significant improvement, i.e., a decrease in pain and an increase in mouth opening. The single-puncture technique provided similar pain reduction as the two-needle approach but provided significantly better results in terms of maximum mouth opening. The reduction in pain was similar when comparing the OA and DD cohorts; however, patients with disc displacement achieved significantly better mouth opening than OA. Intracapsular application of medication contributed to a significant decrease in pain in both HA and PRP groups, with platelet-rich fibrin being significantly superior to HA in terms of mouth opening improvement.

## 1. Introduction

Temporomandibular joint disorders (TMDs) are a group of complex and diversified clinical conditions impairing the physiological functioning of the masticatory system. The American Academy of Orofacial Pain classifies TMDs as a group of disorders involving the masticatory muscles, the temporomandibular joint (TMJ), and the associated structures. These may be caused by abnormal biomechanical forces in the TMJ, injuries, acquired or congenital deformations, a lack of lubrication, degenerative articular disorder, malocclusion, joint hypermobility, and weakness or laxity of the TMJ ligament and joint capsule [1,2]. Recently, short sleep has been discussed as another possible causal factor of TMDs [3]. Genetic factors such as polymorphisms in the genes related to pain perception, inflammation, bone metabolism, and neurotransmission and their significance in the onset and development of TMDs are still being investigated [4].

In clinical conditions, common complaints among patients are myalgia, involving masticatory muscle pain with or without complications, and arthralgia associated with TMJ pain and headaches. This may lead to sequela and other unfavorable consequences. If left untreated, TMDs may contribute to limitations in jaw functionality and thus in oral health and quality of life [5]. Patients with temporomandibular disorders has been proven to exhibit causal association with depression [6]. Moreover, it may lead to sleep deprivation, oral parafunction, stress, anxiety, and somatization [7].

Current treatment protocols assume an interdisciplinary approach. Depending on the severity of joint dysfunction, it may be limited to conservative approaches such as rehabilitation supplemented with dental treatment, splints, orthodontics, physical therapy, and psychological approaches [8]. More advanced techniques assume the use of nerve blocks and surgical procedures. Arthrocentesis of the temporomandibular joints was first described by Nitzan et al. in 1991 [9]. Since that moment, there have been many modifications introduced, mostly comparing Nitzan’s original double-needle technique with the single-puncture needle technique or other modifications [10,11]. Arthrocentesis has been proven to bridge the gap between conservative and surgical approaches to TMDs’ treatment protocol.

Additional use of the intracapsular injection of medicines has been analyzed and compared in many studies, such as with hyaluronic acid (HA), corticosteroids (CS), bone marrow concentrate (BMAC), injectable platelet-rich fibrin (i-PRF), concentrated growth factor (CGF), tenoxicam (TX), micro-fragmented adipose tissue (FAT), and their combined regimens [12,13,14]. Nevertheless, arthrocentesis may decrease the severity of TMDs and limit the necessity of more invasive treatments such as arthroscopy, open disc repositioning, or total arthroplasty with the use of TMJ prosthesis. The aim of this study was to evaluate the efficiency of arthrocentesis in a cohort of patients suffering from TMDs, to evaluate which technique is more efficient, and to justify the injection of additional medicine into the joints and describe their impact on the overall treatment outcome.

## 2. Materials and Methods

A retrospective clinical control study of patients who presented with TMJ arthrocentesis and were diagnosed with TMDs in the Department of Cranio-Maxillofacial Surgery, Oral Surgery and Implantology, Medical University of Warsaw, Poland, was performed. All patients were diagnosed clinically and radiographically with informed consent prior to the procedure. The study was performed in accordance with the Declaration of Helsinki, and the approval of the ethics committee was obtained for the study.

The patients who met the following criteria were selected for the study, and patients were classified according to our modified Wilkes classification system (Table 1): clinical presentation of pain in the TMJ region on movement of the mandible; restriction of mouth opening; clicking during mandibular movements; pain; deviation of mandible on opening the mouth; regular checkups; and completed 6 sessions of arthrocentesis performed every month according to the protocol used in the Department of Cranio-Maxillofacial Surgery, Oral Surgery and Implantology, Medical University of Warsaw, Poland.

The patients who did not meet the following criteria were excluded from the study: lack of informed consent; patients who suffered muscular pain without diagnosis of TMDs; indications for arthroscopy or other invasive surgical treatment; medically compromised patients; incomplete documentation; or fewer than 6 sessions of TMJ arthrocentesis performed.

**Figure 1:**
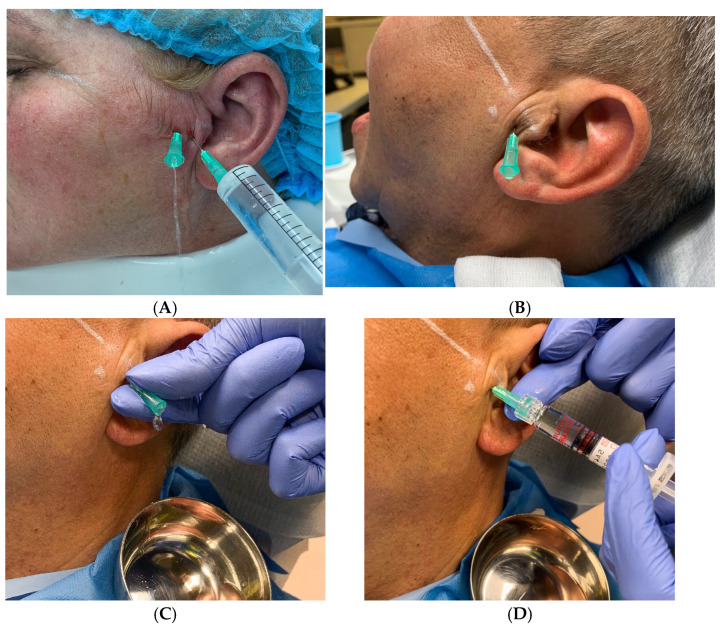
Intraoperative photographs showing two arthrocentesis techniques: (**A**) a two-needle approach where fluid is introduced into the TMJ through a posterior entry point and evacuated simultaneously through an anterior exit point, which provides passive joint lavage; (**B**,**C**) a single puncture technique where fluid is pumped into the TMJ through a posterior point and then evacuated through the same needle after removal of the syringe, which provides a pressured lavage through joint capsule hydro-dissection and stretching; (**D**) intracapsular application of medication after arthrocentesis (HA or PRF).

After informed consent was obtained, 96 patients were randomly assigned to either the single-puncture or the double-needle group. After local anesthesia, TMJ arthrocentesis, consisting of joint lavage using 25–60 mL of lactated Ringer solution, was performed by a single investigator. The two-needle arthrocentesis procedure was performed according to the original technique described by Nitzan [15]. The patient was seated inclined at a 45° angle with their head turned towards the unaffected side to provide access to the appropriate joint. A line was drawn from the middle of the tragus to the outer canthus. The points of insertion were marked on the skin according to McCain points used in arthroscopy [16]. The posterior entrance point was located along the canthotragal line, 10 mm from the middle of the tragus and 2 mm below the line (Figure 1). The anterior point of entry was placed 10 mm farther along the line and 10 mm below it. These markings over the skin indicate the location of the articular fossa and the eminence of the TMJ (Figure 1). In the single-needle puncture pumping technique, only a posterior access point was used for joint lavage according to the technique described by Murakami et al. (1987) [11]. Further, 96 patients were randomly assigned to either the hyaluronic acid (HA) or platelet-rich fibrin (PRF) groups, both of which were infused into the superior joint space. Through posterior access, 1 mL of hyaluronic acid (Biovico, Biolevox HA 2.2%, Gdynia, Poland) or 1 mL of platelet-rich plasma (PRF) was injected into the upper compartment of the TMJ. Pain assessment was conducted using the traditional paper-based VAS assessment scale (VAS), rated 1–10 [17]. Mouth opening was measured using a caliper. The study subjects were followed up every month for 7 months, and assigned repeated protocol data were recorded concerning any reduction in pain and improvement in maximum mouth opening. The individuals evaluating the post-treatment outcomes were separately calibrated, blinded examiners. The statistician was blinded in this study to prevent bias.

The outcomes of the treatment were graded as good, acceptable, or insufficient (failure) in accordance with Table 2, as previously described [18,19].

The data obtained in the study were analyzed using IBM SPSS Statistics for Windows, Version 22.0 IBM Corp. A significance level with a *p*-value < 0.05 was considered statistically significant.

## 3. Results

A total of 96 patients who attended TMJ arthrocentesis were enrolled into the study. Of these, 48 patients were diagnosed with established osteoarthritis (OA) (50%) and 48 with TMJ internal de-arrangement (disc displacement (DD)). All individuals underwent TMJ arthrocentesis with either the one-needle or two-needle technique performed every 4 weeks during a 6-month treatment period. Intracapsular application of the medicine (PRP or HA) was performed in 81 individuals. A total of 15 patient refused intracapsular injection (Table 3).

**Figure 2:**
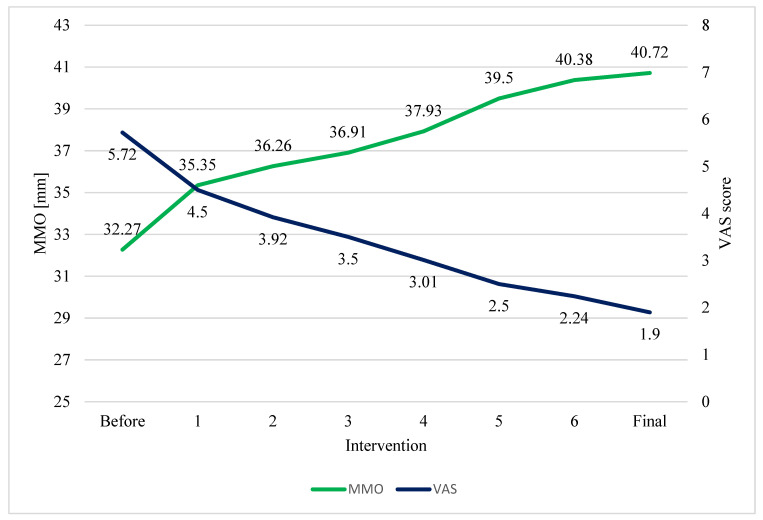
Change in mean VAS and MMO scores (N = 96)**.** A one-way analysis of variance (ANOVA) with repeated measures showed a significant difference between the variables: F = 205.36, *p*-value ≤ 0.001 for the VAS decrease and F = 91.54, *p*-value ≤ 0.001 for increase in MMO before and after the treatment.

Patients who underwent six cycles of arthrocentesis exhibited statistically significant pain reduction and increased mouth opening at the endpoint of the study (Table 3) and after each intervention, as shown by the results of the analysis of variance (ANOVA) of variables VAS and MMO at different time points (Table 4) (Figure 2).

The single-puncture technique provided similar pain reduction as the two-needle approach. The VAS decreases of mean 3.65 ± 1.25 for the single-needle technique and 4.11 ± 1.21 for the two-needle technique were observed without statistically significant differences in paired t-tests (*p* = 0.008). However, the single-puncture technique provided better results in maximum mouth opening of 9.98 ± 6.52 (*p* < 0.001). The two-needle technique provided 4.97 ± 3.97 mm improvement (Table S1).

**Figure 3:**
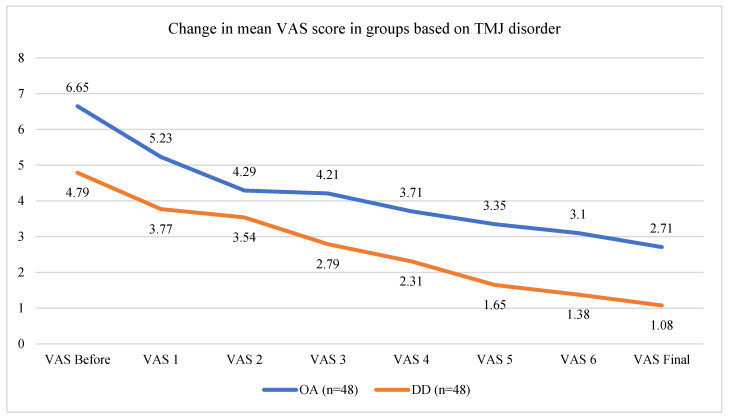
Graph showing gradual decrease in pain between each treatment in the OA and DD cohorts.

**Figure 4:**
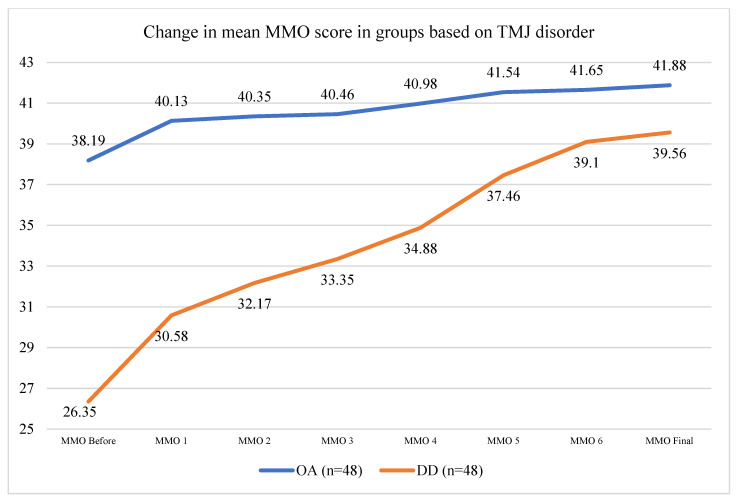
Graph showing gradual increase in maximal mouth opening between each treatment in the OA and DD cohorts.

Patients with osteoarthritis (OA group) exhibited higher VAS scores and more limited mouth opening than patients with disc displacement (DD group) before the treatment (Table S2) (Figure 3). The OA group’s mean age was 56.46 ± 11.93 years old, whereas the DD group was characterized by younger individuals (with a mean age of 30.71 ± 8.13). Both groups exhibited statistically significant pain reduction and increased mouth opening at the endpoint of the study (Figure 2). Reductions in pain were similar when comparing between the groups (*p* = 0.371). However, patients with DD reached significantly better mouth opening than the OA group at the endpoint of the study in paired t-tests (increase of 12.75 ± 4.65 mm vs. increase of 3.46 ± 3.39) (*p* < 0.001) (Table S2) (Figure 4).

**Figure 5:**
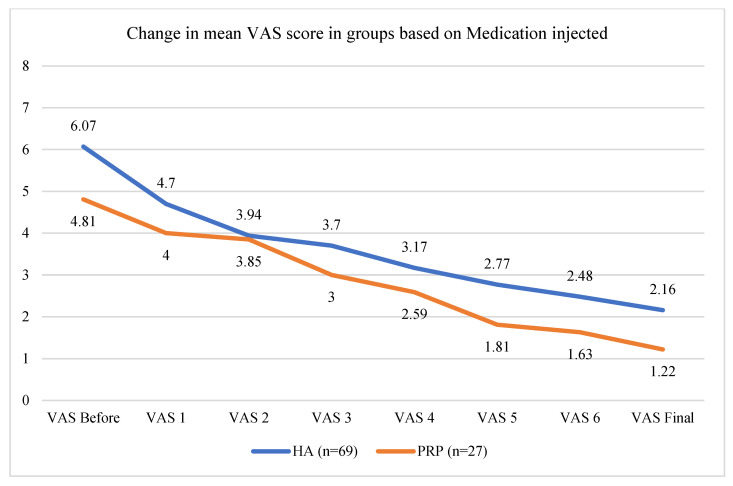
Graph showing gradual decrease in pain between each treatment in the HA and PRP cohorts.

**Figure 6:**
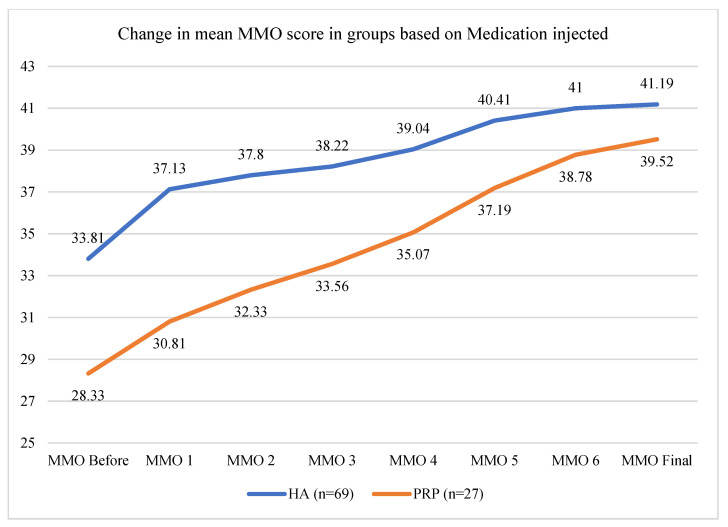
Graph showing gradual increase in maximal mouth opening between each treatment in the HA and PRP cohorts.

Intracapsular application of medication contributed to a decrease in pain in both the HA and PRP cohorts. The mean VAS decrease at the endpoint of the study was 3.91 ± 1.21 for the HA group and 3.59 ± 1.34 for the PRP group, respectively, showing a similar impact on pain reduction associated with TMDs (DD or OA) without statistically significant differences in paired t-tests (*p* = 0.026) (Figure 5). Mouth opening at the endpoint of the study was improved in both cohorts (Figure 6). However, PRP injection was significantly more efficient than HA (*p* = 0.019). The mean final increase in maximal mouth opening in the PRP cohort was improved by 10.44 ± 5.18 mm, whereas in the HA cohort, it was improved by 7.19 ± 6.33 mm (Figure 6).

Twenty patients were subjected to more invasive surgical treatment arthroscopy. In this cohort, the mean VAS level after the completion of six cycles of arthrocentesis remained at the level of mean 3.55 ± 1.88, showing constant or moderate pain despite a statistically significant VAS decrease from mean 6.55 ± 1.79. This cohort also exhibited a worse MMO increase from mean 31.65 mm ± 7.8 mm to 37.1 ± 4.94 mm, which gave a mean of 5.35 ± 6.07 mm improvement. A total of 76 patients were classified as having “good” or “acceptable” treatment results (79.16%). In this cohort, a mean VAS of 1.46 ± 1.29 and a mean MMO of 41.67 ± 3.22 mm were reported at the endpoint of the study, showing significant clinical improvement (Table S3).

## 4. Discussion

Temporomandibular disorders (TMDs) are a complex group of musculoskeletal and neuromuscular conditions that involve the temporomandibular joint. Internal derangements of the TMJ are a result of altered or disturbed movement of the mandibular condyle in the glenoid fossa or against the articular disk. The TMJ may also be affected by osteoarthritis (degenerative joint disease), impaired by condylar hyper- or hypoplasia, or associated with temporomandibular myofascial pain syndrome [20,21,22]. Regardless of the causative effect, pain and limited mouth opening are the most common factors for which patients are referred to a specialist. Arthrocentesis is a procedure performed to collect synovial fluid from joint spaces for the identification of a disease process or the relief of painful or bothersome symptoms [23]. Arthrocentesis of the temporomandibular joints has been widely established as a tool in the minimally invasive treatment of joint disorders since its introduction by Nitzan et al. in 1991 [9]. The aim of a treatment is to reduce pain and increase mouth opening as these are the most morbid factors associated with temporomandibular disorders (TMDs). Currently, there are two main techniques established. One, originally described by Nitzan et al. (1991), where TMJ is lavaged through a two-needle approach, and a second technique introduced by Sing and Varghese (2013), where a single needle is applied. Both techniques have been compared with respect to the decrease in pain and increase in mouth opening. Also, until now, different modifications and approaches have been reported by many authors, including joint lavage techniques, different volumes of fluid used, the application of different medications into the capsular space, the number of sessions and their intervals, and more. Moreover, these studies are characterized by significant heterogeneity, bias in randomization, allocation concealment, and blinding, which make the literature seems inconclusive [24,25,26,27,28,29].

However, data pulled from recent extensive systematic reviews with meta-analyses showed that both techniques (single puncture or double-needle) provided comparable results in terms of pain decrease and mouth opening increase [30,31,32].

Navaneetham et al. (2023) indicated that since there were no statistically significant differences between the single- or double-needle techniques in terms of clinical improvement, one should use a single-puncture approach due to its shorter performance duration, easier execution, and less-invasive nature when compared to the two-needle approach to reduce the risk of nerve damage [33]. Similar observations were described in the studies of Nagori et al. (2020) and Grossman and Poluha (2022) [34,35]. In our study, however, the single-puncture technique provided better maximal mouth opening than the two-needle technique. This could be a result of the pressure-generated distension of the upper compartment of the TMJ and the better impact of the fluid-generated enlargement of the joint space, the increased intra-articular pressure, the removal of adhesions and adherences, and the alterations to the synovial fluid viscosity, which was previously discussed in the study of Grossman et al. (2017) [24]. However, different studies may vary, and our observation is not a rule when comparing our study with those of other researchers due to considerable heterogeneity of the research regarding the treatment of TMDs treatment [29].

Multiple sessions of arthrocentesis were proven to be more effective in terms of clinical outcomes in an extensive systematic review by Guarda-Nardini et al. (2021). The authors proved that three to five sessions are superior than single session. This was also confirmed in an earlier clinical study by Kutuk et al. (2019). These findings were the reason for the further treatment protocol applied in this study which confirmed the gradual clinical improvement between six consecutive monthly sessions at the endpoint of the study. However, fewer than 25% individuals remained symptomatic and were referred for additional treatment, such as arthroscopy, which is consistent with the congruent literature [24].

Additional joint injection strategies were proposed in order to enhance the effect of arthrocentesis, such as use of hyaluronic acid (HA), platelet-rich fibrin/plasma, corticosteroids, mesenchymal stem cells, or others [14]. Despite the fact that HA or PRF/PRP, when used alone, i.e., without arthrocentesis, did not show a superior effect to arthrocentesis alone, they are the most commonly used adjuvants in joint lavage procedures [30].

Hyaluronic acid is a crucial component of the extracellular matrix; it is responsible for the viscoelastic properties of joints. It plays an important role in cell growth and the chondrogenic differentiation of stem cells, binding sites for growth factors and tissue healing. It has also been shown that HA formulations may decrease pro-inflammatory cytokine levels and metalloproteinases [14,36,37]. Platelet-rich fibrin (PRF) or plasma (PRP) was proven to have a beneficial effect on tissue regeneration through growth factors such as TGFβ, IGF, VEGF, PDGF (platelet-derived growth factor), and bFGF (basic fibroblast growth factor). These, present in PRP/PRF, promote the proliferation of chondrogenic cells and the secretion of cartilaginous matrix components [37].

Clinically, HA and PRP were shown to be comparable in terms of pain reduction and increase in mouth opening in the recently published literature. In the study of Jacob et al. (2021), PRP injections provided pain reduction of a 3.79 and 6.52 mm MMO increase, whereas HA provided a VAS reduction of 3.34 and an MMO increase of 11.6 mm. In another study, Kilic et al. (2016) found a mean VAS change of 4.68 for the PRP group and 5.17 for HA group [38].

In our research, a mean VAS change of 3.91 ± 1.21 for HA and 3.59 ± 1.34 for PRP was found.

The mean final increase in maximal mouth opening in the PRP cohort was improved by 10.44 ± 5.18 mm, whereas in the HA cohort, it was by 7.19 ± 6.33 mm. PRF was significantly more efficient in increasing maximal mouth opening.

Despite the overall success of the arthrocentesis, regardless of its type or the medication applied, according to Table 1, in 20 patients, treatment was insufficient according to Angleo et al. (2024) and Eriksson and Westesson (2001); hence, patients qualified for surgical treatment [18,19].

Based on the results described within this article, as well as recent updates in TMJ treatment concepts, arthrocentesis with or without the injection of the medicine into the capsular space should be considered as soon as possible when a conservative approach does show a clear benefit. It offers significant pain reduction and enables wider mouth opening in more than 70% of patients with diagnosed OA or DD.

## 5. Conclusions

Account for the limitations of this retrospective control study with large heterogenicity in the enrolled cohort, it may be concluded that both techniques are equally efficient in the reduction of pain; however, the single-puncture approach proved to be more effective in increasing mouth opening. Multiple sessions of arthrocentesis are advised, preferably six and performed monthly, as clinical improvement is not linear. The intracapsular injection of PRP or HA has a similarly beneficial effect in terms of pain reduction. However, in this study, PRP appeared to be superior to HA in increasing maximal mouth opening.

## Figures and Tables

**Table 1:** Wilkes classification of TMJ internal derangement with modified assignment to the one of the following two groups: disc displacement and osteoarthritis. The reason for the modification was to evaluate weather arthrocentesis is more efficient in the early (structural changes in the bones absent) or late stages (structural changes in the bones present).

Stage	Clinical Symptoms	Radiological Imaging	Group Assignment in the Study
1. Early	Painless clicking; unrestricted function	Mild disc displacement; normal condyle	Disc displacement (DD)
2. Early/Intermediate	Intermittent painful clicking and locking	Mild disc displacement and deformity; normal condyle	
3. Intermediate	Frequent joint pain and locking; painful restricted function; chewing difficulties	Moderate disc displacement and deformity; normal condyle	
4. Intermediate/Late	Chronic pain and restricted mandibular function	Severe disc displacement and deformity; abnormal condyle	Osteoarthritis (OA)
5. Late	Severe joint dysfunction (crepitus) with variable pain	Severe disc displacement with perforation and deformity; degenerative condylar changes	

**Table 2:** Criteria for intervention outcome.

	VAS (on 0–10 Scale)	MMO
Good	No pain or only mild pain: level ≤ 2	≥35 mm
Acceptable	No pain or only mild pain: level ≤ 2	≥30 mm and <35 mm
Insufficient	Constant or moderate pain: level > 2	<30 mm

**Table 3:** Main characteristics of the studied sample (N = 96).

		Frequency	%
TMJ disorder	OA	48	50%
	DD	48	50%
Medication injected	HA	69	71.88%
	PRP	27	28.13%
Treatment	1-needle	60	62.50%
	2-needle	36	37.50%
Intracapsular medication application	yes	81	84.38%
	no	15	15.63%
Further surgical treatment—arthroscopy	yes	20	20.83%
	no	76	79.17%

**Table 4:** Average values and standard deviations of the characteristics of the studied sample (N = 96).

	Mean	Std. Deviation	Minimum	Maximum	Paired t-Test Results (Before and After the Treatment)
Age	43.58	16.45	18	87	
Volume of fluid used (mL)	37.6	11.9	25	60	
VAS Before	5.72	1.8	2	9	t(96) = 15.286 *p* < 0.001
VAS Final	1.9	1.66	0	8	
MMO Before	32.27	8.11	18	46	t(96) = −9.126 *p* < 0.001
MMO Final	40.72	4.07	26	48	

## Data Availability

The original contributions presented in the study are included in the article/Supplementary Material, further inquiries can be directed to the corresponding author.

## Supplementary Materials

The following supporting information can be downloaded at https://www.mdpi.com/article/10.3390/life14121594/s1. File S1. characteristics of the patients enrolled inbto the study; Statistical Analysis: Table S1. Comparative analysis of all sample characteristics according to Treatment, N = 96; Table S2. Comparative analysis of all sample characteristics according to TMJ disorder, N = 96; Table S3. Comparative analysis of all sample characteristics according to Further surgical treatment—arthroscopy, N = 96.
