# Supplementary material for: Arthrocentesis of Temporomandibular Joints—A Clinical Comparative Study
Source: Life (Basel). 2024 Dec 3;14(12):1594. doi: 10.3390/life14121594 (PMC11679531; doi:10.3390/life14121594)
Supplement: Supplementary file 1 [file life-14-01594-s001.zip › Suppl. data.- statistical analysis.pdf]

Table S1. Comparative analysis of all sample characteristics according to Treatment, N=96

	1-needle (n=60)		2-needle (n=36)		t-test for independent samples
	Mean	Std. Deviation	Mean	Std. Deviation	p-value
Age	38.82	16.52	51.53	13.07	<0.001
Volume of fluid used (ml)	33.42	10.99	44.58	10.03	<0.001
VAS Before	5.12	1.72	6.72	1.49	<0.001
VAS 1	4.05	1.53	5.25	1.4	<0.001
VAS 2	3.58	1.42	4.47	1.34	0.003
VAS 3	2.92	1.52	4.47	1.59	<0.001
VAS 4	2.45	1.71	3.94	1.55	<0.001
VAS 5	1.85	1.46	3.58	1.83	<0.001
VAS 6	1.77	1.53	3.03	1.78	<0.001
VAS Final	1.47	1.51	2.61	1.66	0.001
MMO Before	29.73	8.27	36.5	5.8	<0.001
MMO 1	33.27	6.94	38.83	5.32	<0.001
MMO 2	34.48	6.22	39.22	4.76	<0.001
MMO 3	35.48	6.01	39.28	5	0.002
MMO 4	37.22	5.56	39.11	7.65	0.165
MMO 5	38.53	4.63	41.11	3.88	0.006
MMO 6	39.72	4.4	41.47	3.54	0.045
MMO Final	40.12	4.27	41.72	3.53	0.061
Δ VAS	3.65	1.25	4.11	1.21	0.08
Δ MMO	9.98	6.52	4.97	3.97	<0.001

Table S2. Comparative analysis of all sample characteristics according to TMJ disorder, N=96

	OA (n=48)		DD (n=48)		t-test for independent samples
	Mean	Std. Deviation	Mean	Std. Deviation	p-value
Age	56.46	11.93	30.71	8.13	<0.001
Volume of fluid used (ml)	45.1	9.97	30.1	8.47	<0.001
VAS Before	6.65	1.28	4.79	1.79	<0.001
VAS 1	5.23	1.29	3.77	1.53	<0.001
VAS 2	4.29	1.32	3.54	1.49	0.01
VAS 3	4.21	1.53	2.79	1.61	<0.001
VAS 4	3.71	1.46	2.31	1.85	<0.001
VAS 5	3.35	1.69	1.65	1.49	<0.001
VAS 6	3.1	1.57	1.38	1.44	<0.001

VAS Final	2.71	1.43	1.08	1.47	<0.001
MMO Before	38.19	5.56	26.35	5.51	<0.001
MMO 1	40.13	5.01	30.58	4.97	<0.001
MMO 2	40.35	4.44	32.17	4.71	<0.001
MMO 3	40.46	4.8	33.35	4.69	<0.001
MMO 4	40.98	4.55	34.88	6.66	<0.001
MMO 5	41.54	3.93	37.46	4.16	<0.001
MMO 6	41.65	3.98	39.1	4	0.002
MMO Final	41.88	3.85	39.56	3.99	0.005
Δ VAS	3.94	1.08	3.71	1.4	0.371
Δ MMO	3.46	3.39	12.75	4.65	<0.001

Table S3. Comparative analysis of all sample characteristics according to Further surgical treatment - arthroscopy, N=96

	yes (n=20)		no (n=76)		t-test for independent samples
	Mean	Std. Deviation	Mean	Std. Deviation	p-value
Age	43	15.81	43.74	16.71	0.86
Volume of fluid used (ml)	40	13.28	36.97	11.52	0.314
VAS Before	6.55	1.79	5.5	1.75	0.02
VAS 1	4.95	1.7	4.38	1.55	0.156
VAS 2	4.65	1.27	3.72	1.44	0.01
VAS 3	4.35	1.46	3.28	1.72	0.012
VAS 4	4.4	1.5	2.64	1.69	<0.001
VAS 5	3.6	1.88	2.21	1.68	0.002
VAS 6	3.85	1.98	1.82	1.39	<0.001
VAS Final	3.55	1.88	1.46	1.29	<0.001
MMO Before	31.65	7.8	32.43	8.23	0.702
MMO 1	35.15	6.43	35.41	7.06	0.883
MMO 2	35.4	6.06	36.49	6.18	0.484
MMO 3	35.15	5.49	37.37	5.98	0.137
MMO 4	35.9	5.53	38.46	6.6	0.115
MMO 5	36.95	4.83	40.17	4.22	0.004
MMO 6	37	4.84	41.26	3.5	<0.001
MMO Final	37.1	4.94	41.67	3.22	<0.001
Δ VAS	3	1.59	4.04	1.05	0.001
Δ MMO	5.35	6.07	8.83	6.04	0.024
